# Comparative Evaluation of Salivary Zinc Finger E-Box Binding Homeobox 1 (ZEB1) Levels in Healthy Individuals, Periodontitis, and Oral Squamous Cell Carcinoma Patients: A Cross-Sectional Study

**DOI:** 10.7759/cureus.114325

**Published:** 2026-08-11

**Authors:** Saniya A Shikalgar, Sameer A Zope, Girish Suragimath, Siddhartha Varma, Apurva V Kale, Rashmi A Gudur

**Affiliations:** 1 Periodontology, School of Dental Sciences, Krishna Vishwa Vidyapeeth (Deemed to be University), Karad, IND; 2 Oncology, Krishna Institute of Medical Sciences, Krishna Vishwa Vidyapeeth (Deemed to be University), Karad, IND

**Keywords:** biomarker, elisa, emt, oral squamous cell carcinoma, periodontitis, saliva, zeb1

## Abstract

Background

Periodontitis and oral squamous cell carcinoma (OSCC) are chronic conditions sharing common pathogenic mechanisms, including inflammation and molecular dysregulation. Zinc finger E-box binding homeobox 1 (ZEB1), a key regulator of epithelial-mesenchymal transition (EMT), may serve as a potential biomarker linking these conditions. This study aimed to evaluate and compare salivary ZEB1 levels and clinical periodontal parameters among healthy individuals, periodontitis patients, and patients with oral squamous cell carcinoma (OSCC), and to assess the potential of salivary ZEB1 as a non-invasive biomarker associated with periodontal disease and oral cancer.

Methodology

A total of 90 participants were included in this cross-sectional study. The patients were categorized into the following three equal groups: Group A included healthy individuals, Group B included periodontitis patients, and Group C included OSCC patients. Periodontal parameters, gingival index, plaque index, probing pocket depth, and clinical attachment loss were recorded in Groups A and B. Unstimulated saliva samples were obtained from all participants and subjected to enzyme-linked immunosorbent assay analysis for the estimation of ZEB1 levels. Statistical evaluation of the data was performed using one-way analysis of variance, followed by Tukey’s post hoc test.

Results

The periodontitis group demonstrated significantly higher clinical periodontal parameters (p < 0.001). Salivary ZEB1 levels were significantly higher across the groups: Group A (3.28 ± 1.72 ng/mL), Group B (12.11 ± 6.81 ng/mL), and Group C (14.96 ± 6.24 ng/mL) (p < 0.001). Intergroup comparisons revealed statistically significant differences between all groups. Elevated ZEB1 levels were associated with increased disease severity.

Conclusions

Salivary ZEB1 levels were significantly elevated in patients with periodontitis and OSCC, with the highest levels observed in the OSCC group. These findings suggest that ZEB1 may act as a promising non-invasive biomarker linking periodontal inflammation and oral carcinogenesis. It may also have potential applications in early detection and disease monitoring.

## Introduction

Oral health is closely associated with overall systemic health, and oral diseases may reflect underlying systemic conditions. Periodontitis and oral squamous cell carcinoma (OSCC) are major oral diseases characterized by high prevalence, chronic progression, and significant impact on quality of life [1]. Although etiologically distinct, both conditions share mechanisms such as chronic inflammation, tissue destruction, and molecular dysregulation, suggesting a possible biological link [2].

Periodontitis is a chronic inflammatory condition of the tooth-supporting tissues, initiated by microbial biofilm accumulation and mediated by an altered host immune response [3]. This leads to the release of pro-inflammatory mediators, matrix metalloproteinases, and reactive oxygen species, resulting in connective tissue breakdown and alveolar bone loss. Growing evidence highlights its systemic associations, mediated by persistent low-grade inflammation and the dissemination of bacterial products, linking it to conditions such as cardiovascular diseases, diabetes, and malignancies [4].

OSCC accounts for the majority of oral cancers and remains a global health burden, particularly in individuals exposed to risk factors such as tobacco, alcohol, and poor oral hygiene. Its pathogenesis involves cumulative genetic and epigenetic alterations, with chronic inflammation playing a key role in tumor initiation and progression [5].

Epithelial-mesenchymal transition (EMT) plays a pivotal role in cancer progression by facilitating the transformation of epithelial cells into a more invasive phenotype, thereby contributing to tumor spread and metastatic potential. Zinc finger E-box binding homeobox 1 (ZEB1), a key EMT-regulating transcription factor, promotes tumor progression by suppressing epithelial markers such as E-cadherin, Claudin-4, and occludin, while upregulating mesenchymal markers including vimentin and fibronectin-1, thereby enhancing cellular migration, invasion, and disease progression [6]. Emerging evidence suggests that chronic inflammation, as seen in periodontitis, may influence ZEB1 expression, indicating a mechanistic link between periodontal disease and OSCC [7,8]. Based on these observations, we hypothesized that salivary ZEB1 levels would be elevated in patients with periodontitis and OSCC compared with periodontally healthy individuals and would be associated with clinical periodontal parameters.

Saliva offers a non-invasive diagnostic medium reflecting both local and systemic changes. However, to date, few studies have investigated salivary ZEB1 levels in periodontitis or OSCC, and no study has comprehensively compared salivary ZEB1 concentrations among healthy individuals, periodontitis patients, and OSCC patients within a single study population. Therefore, the primary aim of this study was to evaluate and compare salivary ZEB1 levels among healthy individuals, periodontitis patients, and patients with OSCC to assess its potential as a non-invasive biomarker. The secondary aim was to evaluate and compare the periodontal clinical parameters among these groups and explore their association with salivary ZEB1 levels.

## Materials and methods

Study design

This cross-sectional study was conducted in the Department of Periodontology in collaboration with the Department of Oncology at Krishna Vishwa Vidyapeeth, Karad. Before the initiation of the study, all participants were informed about the study objectives and procedures, and written informed consent was obtained from each participant before enrollment. Patients were recruited from June 30, 2024, to July 15, 2025.

Ethical considerations

The study protocol was reviewed and approved by the Institutional Ethics Committee of Krishna Vishwa Vidyapeeth, Karad, before participant recruitment and data collection (IEC No: KVV/IEC/10/2024). The Strengthening the Reporting of Observational Studies in Epidemiology (STROBE) guidelines were used to design, analyze, and interpret this study. The study was conducted in accordance with institutional ethical guidelines and the principles outlined in the Declaration of Helsinki for research involving human participants.

Selection criteria

Patients fulfilling the eligibility criteria and providing written informed consent were recruited for the study. Periodontitis was diagnosed based on the diagnostic criteria defined by the 2017 World Workshop Classification of Periodontal and Peri-Implant Diseases and Conditions. Following a comprehensive periodontal examination and oral screening for oral cancer, the participants were categorized into study groups. A total of 90 subjects aged 20-60 years were screened, and all eligible participants were selected using purposive sampling. The study population was categorized into the following three groups: Group A included periodontally healthy individuals exhibiting no clinical attachment loss (CAL), probing pocket depth (PPD) of ≤3 mm, and bleeding on probing at fewer than 10% of examined sites; Group B included patients with generalized periodontitis (Stage II, III, and IV); and Group C comprised patients diagnosed with OSCC, confirmed through both clinical examination and histopathological evaluation. Subjects with a history of systemic disorders; pregnant or lactating women; individuals who had undergone periodontal therapy within the preceding three months; patients receiving antibiotics, antiplatelet agents, or anti-inflammatory drugs; and individuals undergoing treatment for OSCC were excluded from the study.

Sample size estimation

Sample size estimation was performed using G*Power version 3.0.1 (Franz Faul Universität, Kiel, Germany) using the following formula:

\[ N = \frac{\left(Z_{1-\alpha/2} + Z_{\beta}\right)^2 \left(\sigma_1^2 + \sigma_2^2\right)} {\left(\mu_1 - \mu_2\right)^2} \]

where,

\[ Z_{1-\alpha/2} = \text{standard normal deviate corresponding to the desired confidence level} \]

\[ Z_{\beta} = \text{standard normal deviate corresponding to the desired statistical power} \]

\[ \sigma_1,\ \sigma_2 = \text{standard deviations of the two groups} \]

\[ \mu_1 - \mu_2 = \text{expected difference between the group means} \]

The calculation assumed a large effect size (Cohen’s f = 0.917), a two-sided significance level (α) of 0.05, a statistical power of 95% (1−β = 0.95), and an equal allocation ratio (1:1:1). Based on these assumptions, the minimum required sample size was 90 participants, comprising 30 participants in each study group.

Clinical examination

All clinical periodontal examinations were performed by a single examiner using standardized examination procedures. The clinical parameters recorded included the Simplified Oral Hygiene Index (OHI-S) [9], Russell’s Periodontal Index [10], PPD, and CAL [11]. PPD and CAL were measured using a UNC-15 periodontal probe at six sites per tooth. To minimize measurement variability, all examinations were conducted by the same examiner throughout the study.

Sample collection

A total of 2 mL of saliva was collected between 10:00 AM and 12:00 PM using a modified Navazesh protocol [12]. Participants were instructed to avoid eating, drinking, and oral hygiene practices for at least one hour before sample collection. To avoid blood contamination, clinical examination was completed at least one hour before sampling. Saliva was allowed to accumulate and was passively collected into sterile containers over five minutes. Samples were centrifuged at 1,000 × g for 15 minutes at 2-8°C immediately after collection. The supernatant was separated, aliquoted, and stored at −80°C until analysis. All analyses were performed within two months to preserve biomarker stability.

Biochemical examination

All saliva samples were coded before analysis, and the laboratory personnel conducting the enzyme-linked immunosorbent assay (ELISA) assays were blinded to the clinical group assignments of the participants. Each aliquot was thawed only once at the time of ELISA analysis, and repeated freeze-thaw cycles were avoided to minimize protein degradation and preserve biomarker stability. Salivary ZEB1 levels were quantified using a commercially available ELISA kit (ELK Biotechnology, Wuhan, China) based on the sandwich enzyme immunoassay principle. Standards and test samples were placed into antibody-coated microplate wells. A biotin-labeled detection antibody and streptavidin-horseradish peroxidase solution were then added, followed by incubation and washing procedures. After adding the TMB substrate, the color reaction was stopped with sulfuric acid. Sample absorbance was determined at 450 nm using a microplate reader, and the corresponding concentrations were estimated by interpolation from a standard calibration curve.

The assay employed a seven-point standard curve ranging from 0.16 to 10 ng/mL. Samples yielding concentrations above the upper limit of the standard curve were appropriately diluted with the manufacturer’s sample diluent and re-assayed, and the measured concentrations were multiplied by the corresponding dilution factor to obtain the final concentration. All samples were analyzed in duplicate, and concentrations were calculated from the standard curve generated at 450 nm. According to the manufacturer, the assay demonstrated an intra-assay coefficient of variation (CV) <8%, an inter-assay CV <10%, and acceptable linearity following serial dilution (approximately 80%-108% recovery, depending on sample matrix).

Statistical analysis

Statistical analysis was performed using SPSS Statistics version 25.0 (IBM Corp., Armonk, NY, USA). Quantitative variables were expressed as mean ± standard deviation (SD), while categorical variables were summarized as frequencies and percentages. Descriptive statistics for salivary ZEB1 levels were additionally reported as median and interquartile range (IQR) to better characterize data distribution.

Before inferential analyses, the normality of salivary ZEB1 levels within each study group was assessed using the Shapiro-Wilk test, and homogeneity of variances was evaluated using Levene’s test. Comparisons of mean salivary ZEB1 levels and clinical periodontal parameters among the three study groups were performed using one-way analysis of variance (ANOVA). Tukey’s honestly significant difference (HSD) post hoc test was used for pairwise comparisons following significant ANOVA results. Because the study groups were balanced in size, one-way ANOVA was considered appropriate for the primary analysis.

**Figure 1:**
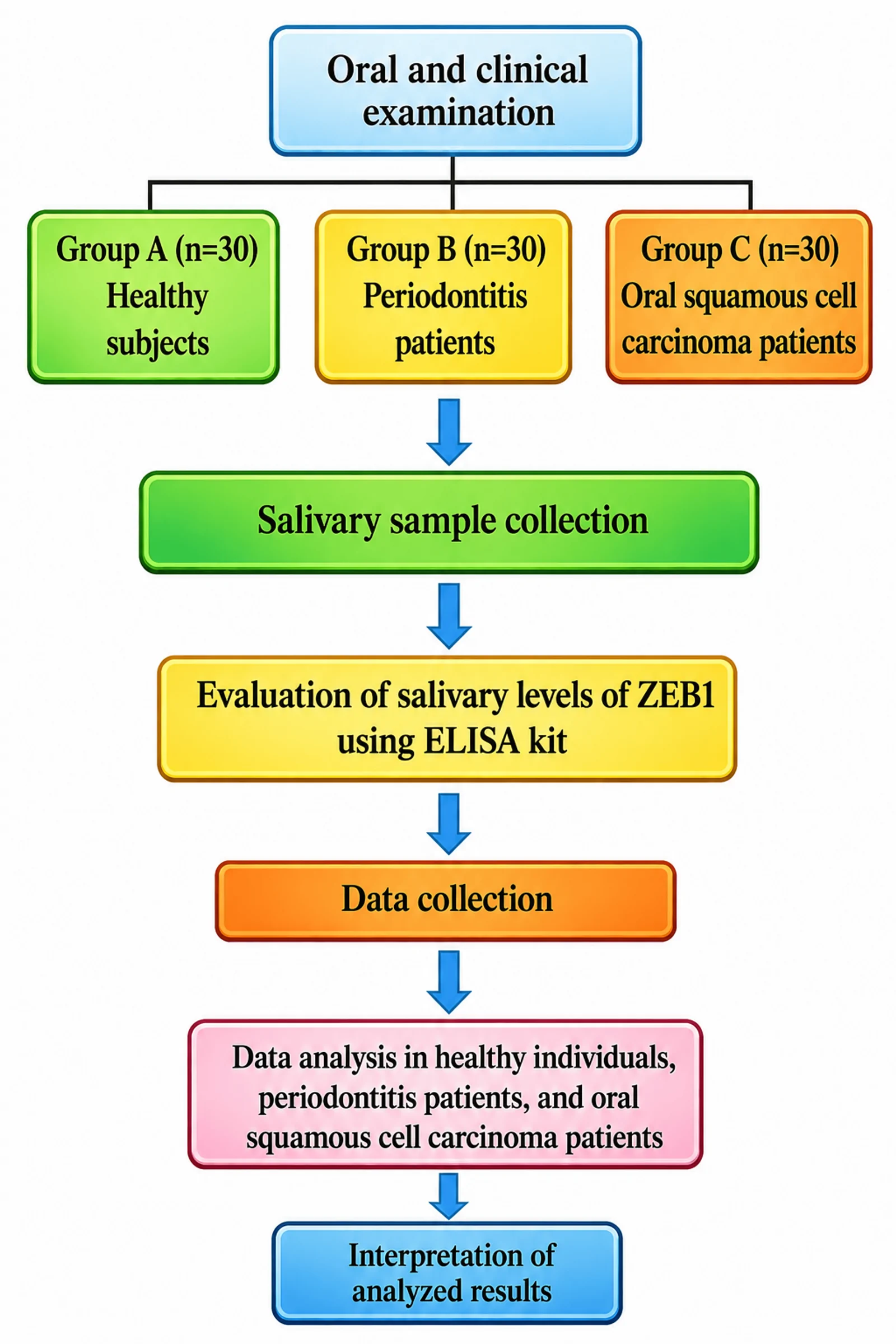
Summary of the study protocol. ZEB1 = zinc finger E-box binding homeobox 1; ELISA = enzyme-linked immunosorbent assay

As Levene’s test indicated unequal variances among the study groups, a Kruskal-Wallis test was additionally performed as a non-parametric sensitivity analysis to confirm the robustness of the intergroup comparisons. To account for the significant difference in age distribution among the study groups, analysis of covariance (ANCOVA) was performed to compare salivary ZEB1 levels after adjusting for age and gender as potential confounding variables. Pearson’s correlation coefficient was used to evaluate the association between salivary ZEB1 levels and clinical periodontal parameters. All statistical tests were two-tailed, and a p-value <0.05 was considered statistically significant. The steps of the study methodology are illustrated in Figure 1.

## Results

The study comprised 90 participants (n = 90) who were equally distributed into three groups (n = 30 in each group): Group A (periodontally healthy individuals), Group B (patients with periodontitis), and Group C (patients with OSCC). Table 1 presents a comparison of mean age among the three study groups. Group A showed a mean age of 40.68 years, whereas Group B demonstrated the highest mean age of 48.40 years. Group C exhibited a mean age of 44.84 years. The intergroup comparison revealed a statistically significant difference in mean age, with an F-value of 3.19 and a p-value of 0.047.

**Table 1:** Comparison of the mean age among the study groups. Values are expressed as mean ± SD. A p-value <0.05 indicates statistical significance. OSCC = oral squamous cell carcinoma; SD = standard deviation; SE = standard error

Group	Mean age ± SD (years)	SE	Range (years)	F-value	P-value
Group A (healthy)	40.68 ± 12.00	2.40	28.68–52.68	3.19	0.047*
Group B (periodontitis)	48.40 ± 8.77	1.75	39.63–57.17		
Group C (OSCC)	44.84 ± 11.40	2.28	33.44–56.24		

Table 2 compares the periodontal clinical parameters (OHI-S, Russell’s Index, PPD, and CAL) among Group A, Group B, and Group C using ANOVA.

**Table 2:** ANOVA test for periodontal clinical parameters. Data are presented as mean ± SD. Statistical analysis was performed using one-way ANOVA, and corresponding F-values are shown in the table. *: A p-value <0.001* was considered statistically significant. OHI-S = Simplified Oral Hygiene Index; PPD = probing pocket depth; CAL = clinical attachment loss; SD = standard deviation; SE = standard error; CI = confidence interval; ANOVA = analysis of variance

Parameters	Group	Mean ± SD	SE	95% CI (lower)	95% CI (upper)	Minimum	Maximum	ANOVA F-value	P-value
OHI-S	Group A	0.548 ± 0.231	0.041	0.464	0.631	0.1	0.9	503.42	<0.001*
	Group B	3.572 ± 0.452	0.081	3.405	3.738	2.8	5.1		
	Group C	2.421 ± 0.398	0.071	2.276	2.566	1.6	3.3		
Russell’s Index	Group A	0.152 ± 0.071	0.013	0.125	0.178	0.0	0.3	569.81	<0.001*
	Group B	3.468 ± 0.362	0.065	3.335	3.601	2.6	4.3		
	Group C	2.789 ± 0.602	0.108	2.568	3.010	1.7	4.1		
PPD	Group A	1.41 ± 0.57	0.041	1.21	1.61	0.5	2.5	472.64	<0.001*
	Group B	7.36 ± 0.832	0.149	7.05	7.67	6	9		
	Group C	6.38 ± 0.901	0.162	6.05	6.71	5	8		
CAL	Group A	0.00 ± 0.000	0.000	0.00	0.00	0	0	196.37	<0.001*
	Group B	4.05 ± 0.902	0.162	3.72	4.38	3	6		
	Group C	3.74 ± 1.142	0.205	3.32	4.16	0	6		

Group A had the lowest mean values for all periodontal parameters, whereas Group B had the highest, followed by Group C. The mean OHI-S scores were 0.548 ± 0.231 in Group A, 3.572 ± 0.452 in Group B, and 2.421 ± 0.398 in Group C, showing a statistically significant difference among the groups (ANOVA F = 503.42, p < 0.001). Similarly, the mean Russell’s Index scores were 0.152 ± 0.071 in Group A, 3.468 ± 0.362 in Group B, and 2.789 ± 0.602 in Group C (ANOVA F = 569.81, p < 0.001).

The mean PPD was 1.41 ± 0.57 mm in Group A, 7.36 ± 0.832 mm in Group B, and 6.38 ± 0.901 mm in Group C, with the difference being statistically significant (ANOVA F = 472.64, p < 0.001). Likewise, the mean CAL was 0.00 ± 0.000 mm in Group A, 4.05 ± 0.902 mm in Group B, and 3.74 ± 1.142 mm in Group C (ANOVA F = 196.37, p < 0.001). Overall, the differences in mean scores of all periodontal parameters among the study groups were statistically highly significant (p < 0.001).

Table 3 presents the intergroup comparison of periodontal clinical parameters using Tukey’s post hoc test following ANOVA analysis. Significant pairwise differences were observed for most parameters among the three groups.

**Table 3:** Tukey’s post hoc analysis of periodontal clinical parameters. Values represent Tukey’s post hoc multiple comparison test following one-way ANOVA. OHI-S (F = 503.42), Russell’s Index (F = 569.81), PPD (F = 472.64), and CAL (F = 196.37) (p < 0.001).* OHI-S = Simplified Oral Hygiene Index; PPD = probing pocket depth; CAL = clinical attachment loss; SD = standard deviation; SE = standard error; CI = confidence interval; ANOVA = analysis of variance

Dependent variable	Reference group	Comparison group	Mean difference	SE	95% CI (lower bound)	95% CI (upper bound)	ANOVA F-value	P-Value
OHI-S	Group A	Group B	-3.024	0.102	-3.235	-2.813	503.42	<0.001*
	Group A	Group C	-1.873	0.095	-2.069	-1.677		<0.001*
	Group B	Group C	1.151	0.089	0.968	1.334		<0.001*
Russell’s Index	Group A	Group B	-3.316	0.087	-3.495	-3.137	569.81	<0.001*
	Group A	Group C	-2.637	0.092	-2.826	-2.448		<0.001*
	Group B	Group C	0.679	0.076	0.523	0.835		0.002*
PPD	Group A	Group B	-5.95	0.168	-6.30	-5.60	472.64	<0.001*
	Group A	Group C	-4.97	0.172	-5.32	-4.62		<0.001*
	Group B	Group C	0.98	0.151	0.67	1.29		0.021*
CAL	Group A	Group B	-4.05	0.182	-4.42	-3.68	196.37	<0.001*
	Group A	Group C	-3.74	0.195	-4.14	-3.34		<0.001*
	Group B	Group C	0.31	0.178	-0.05	0.67		0.882

For OHI-S, Group A showed significantly lower scores than Group B and Group C, with mean differences of -3.024 and -1.873, respectively (p < 0.001). Group B also showed significantly higher scores than Group C, with a mean difference of 1.151 (p < 0.001).

Similarly, Russell’s Index was significantly lower in Group A compared to Group B and Group C, with mean differences of -3.316 and -2.637, respectively (p < 0.001). Group B demonstrated significantly higher scores than Group C, with a mean difference of 0.679 (p = 0.002), indicating greater periodontal destruction.

For PPD, Group A showed significantly shallower pockets than Group B and Group C, with mean differences of -5.95 mm and -4.97 mm, respectively (p < 0.001). Group B also exhibited significantly deeper pockets than Group C, with a mean difference of 0.98 mm (p = 0.021). Regarding CAL, Group A had significantly lower values than Group B and Group C, with mean differences of -4.05 mm and -3.74 mm, respectively (p < 0.001). However, no statistically significant difference was observed between Group B and Group C (mean difference = 0.31 mm, p = 0.882).

Table 4 summarizes the descriptive statistics of salivary ZEB1 levels among the study groups. The healthy group exhibited the lowest salivary ZEB1 concentration, with a mean of 3.28 ± 1.72 ng/mL and a median of 3.35 ng/mL (IQR = 2.52-4.15 ng/mL). In comparison, the periodontitis group demonstrated a marked increase in salivary ZEB1 levels, with a mean of 12.11 ± 6.81 ng/mL and a median of 12.13 ng/mL (IQR = 6.20-15.18 ng/mL). The OSCC group exhibited the highest mean salivary ZEB1 level (14.96 ± 6.24 ng/mL) with a median of 13.90 ng/mL (IQR = 11.31-18.48 ng/mL). The wider IQRs observed in the periodontitis and OSCC groups compared with the healthy group indicate greater variability in salivary ZEB1 levels among diseased participants. Overall, both the mean and median values demonstrated a progressive increase in salivary ZEB1 levels from healthy individuals to patients with periodontitis and OSCC.

**Table 4:** Descriptive statistics of salivary ZEB1 levels among the study groups. Data are presented as mean ± SD, median, and IQR (Q1–Q3). ZEB1 = zinc finger E-box binding homeobox 1; OSCC = oral squamous cell carcinoma; SD = standard deviation; IQR = interquartile range; Q1 = 25th percentile; Q3 = 75th percentile

Group	n	Mean ± SD (ng/mL)	Median (ng/mL)	Q1 (25th Percentile)	Q3 (75th Percentile)	IQR (Q3 − Q1)
Healthy	30	3.28 ± 1.72	3.35	2.52	4.15	1.63
Periodontitis	30	12.11 ± 6.81	12.13	6.20	15.18	8.98
OSCC/Cancer	30	14.96 ± 6.24	13.90	11.31	18.48	7.17

**Figure 2:**
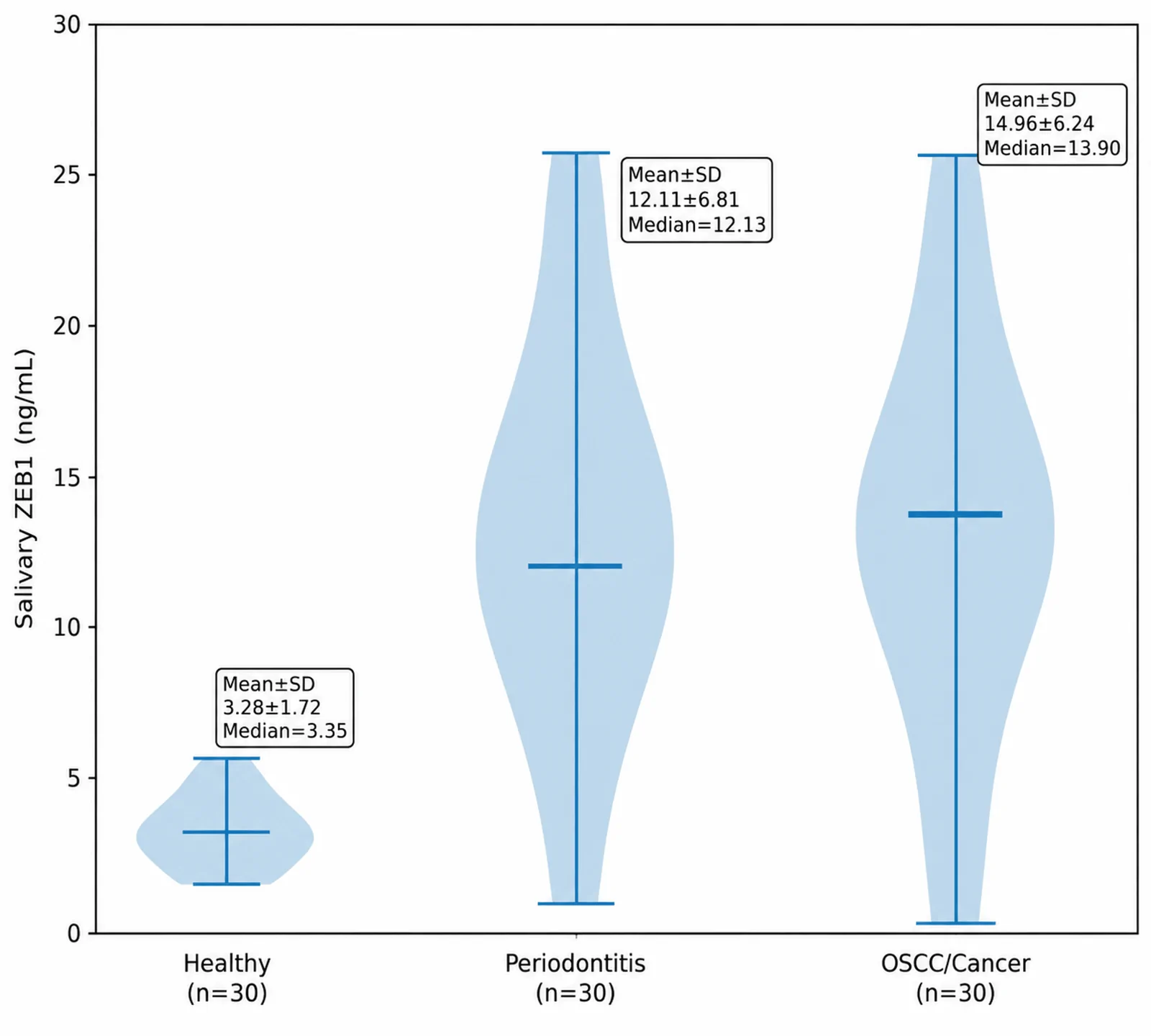
Distribution of salivary ZEB1 levels across the study groups. ZEB1 = zinc finger E-box binding homeobox 1; OSCC = oral squamous cell carcinoma

Figure 2 illustrates the distribution of salivary ZEB1 levels among the study groups.

Table 5 presents the assessment of the statistical assumptions for salivary ZEB1 levels. The Shapiro-Wilk test demonstrated that salivary ZEB1 levels were normally distributed in all three study groups (healthy: W = 0.9751, p = 0.6868; periodontitis: W = 0.9837, p = 0.9130; OSCC: W = 0.9628, p = 0.3654), indicating that the assumption of normality was satisfied. However, Levene’s test revealed a significant violation of the homogeneity of variance assumption (F = 14.7509, p < 0.0001), suggesting unequal variances among the study groups. Therefore, to confirm the robustness of the findings, a non-parametric Kruskal-Wallis test was additionally performed. The Kruskal-Wallis analysis demonstrated a statistically significant difference in salivary ZEB1 levels among the healthy, periodontitis, and OSCC groups (H = 41.8826, df = 2, p < 0.0001), confirming the significant intergroup differences observed in the parametric analysis.

**Table 5:** Assessment of statistical assumptions and non-parametric confirmation for salivary ZEB1 levels among the study groups. Data represent the results of the Shapiro–Wilk test for normality, Levene’s test for homogeneity of variances, and the Kruskal–Wallis test for non-parametric comparison of salivary ZEB1 levels among the healthy, periodontitis, and OSCC groups. Statistical significance was considered at p-values <0.05. ZEB1 = zinc finger E-box binding homeobox 1; OSCC = oral squamous cell carcinoma

Test	Statistic	P-value	Interpretation
Shapiro–Wilk (normality)	Healthy: W = 0.9751; Periodontitis: W = 0.9837; OSCC: W = 0.9628	Healthy: p = 0.6868; Periodontitis: p = 0.9130; OSCC: p = 0.3654	Normal distribution in all groups (p > 0.05)
Levene’s test	F = 14.7509	p < 0.0001	Unequal variances among groups
Kruskal–Wallis test	H = 41.8826 (df = 2)	p < 0.0001	Significant differences confirmed among groups

Table 6 shows statistically significant differences in salivary ZEB1 levels across all study groups, indicating clear intergroup variation. Group C (OSCC patients) exhibited the highest ZEB1 concentrations, with significantly higher levels than Group B (periodontitis) (mean difference = 2.85 ng/mL, p = 0.003) and Group A (healthy) (mean difference = 11.68 ng/mL, p < 0.001). Similarly, Group B (periodontitis) showed significantly higher ZEB1 levels than Group A (healthy), with a mean difference of 8.83 ng/mL (p < 0.001).

**Table 6:** Pairwise comparison of mean salivary ZEB1 levels among the study groups using Tukey’s post hoc test. ZEB1 = zinc finger E-box binding homeobox 1

Comparison between groups	Mean difference (ng/mL)	P-value
Group A vs. Group B	8.83	<0.001
Group A vs. Group C	11.68	<0.001
Group B vs. Group C	2.85	0.003

After adjusting for the potential confounding effects of age and gender, the study group remained a statistically significant predictor of salivary ZEB1 levels (F = 17.155, p < 0.001). This indicates that the differences in ZEB1 levels among the healthy, periodontitis, and OSCC groups were independent of variations in age and gender. In contrast, gender was not significantly associated with salivary ZEB1 levels (F = 0.099, p = 0.753), suggesting that males and females had comparable ZEB1 concentrations after controlling for the other variables. Similarly, age did not have a significant effect on ZEB1 levels (F = 0.586, p = 0.446), indicating that age was not a significant covariate in this model. Overall, these findings demonstrate that the observed differences in salivary ZEB1 levels are primarily attributable to disease status rather than differences in age or gender, supporting the robustness of the study’s main findings after adjustment for these potential confounders (Table 7).

**Table 7:** ANCOVA of salivary ZEB1 levels after adjustment for age and gender. ANCOVA was performed to evaluate differences in salivary ZEB1 levels among the study groups after adjusting for age and gender as potential confounding variables. The table presents the sum of squares, degrees of freedom, F-statistic, and corresponding p-values for each source of variation. Statistical significance was set at p-values <0.05. ZEB1 = zinc finger E-box binding homeobox 1; ANCOVA = analysis of covariance

Source	Sum of squares	df	F-value	P-value
Gender	3.046	1	0.099	0.753
Study group	1,050.474	2	17.155	<0.001
Age	17.942	1	0.586	0.446
Residual	2,602.502	85	—	—

Pearson’s correlation analysis revealed a strong positive correlation between salivary ZEB1 levels and all clinical periodontal parameters. Salivary ZEB1 levels showed significant positive correlations with OHI-S (0.892, p < 0.001), Russell’s Index (0.913, p < 0.001), PPD (0.909, p < 0.001), and CAL (0.888, p < 0.001) (Table 8).

**Table 8:** Pearson correlation analysis between salivary ZEB1 levels and clinical periodontal parameters. Statistically significant at p < 0.05. OHI-S = Simplified Oral Hygiene Index; PPD = probing pocket depth; CAL = clinical attachment loss; ZEB1 = zinc finger E-box binding homeobox 1

Variable	OHI-S	Russell’s Index	PPD	CAL	ZEB1
ZEB1	0.892	0.913	0.909	0.888	1
P-value	<0.001*	<0.001*	<0.001*	<0.001*	—

## Discussion

The present study evaluated salivary ZEB1 levels and periodontal clinical parameters among healthy individuals, periodontitis patients, and patients with OSCC. Significantly higher salivary ZEB1 levels were observed in both periodontitis and OSCC groups compared with healthy controls, with the highest levels detected in OSCC patients. In addition, salivary ZEB1 levels demonstrated significant positive correlations with periodontal clinical parameters, suggesting a potential association between ZEB1 expression, periodontal tissue destruction, and oral carcinogenesis.

Periodontal health depends on a balanced interaction between the host immune response and oral microbiota. Disruption of this balance results in dysbiosis and an increase in pathogenic microorganisms, including *Porphyromonas gingivalis*, *Tannerella forsythia*, and *Fusobacterium nucleatum* [13]. These microorganisms induce chronic inflammation through cytokines such as interleukin (IL)-1β, IL-6, and tumor necrosis factor-alpha, contributing to periodontal tissue destruction [14]. Persistent inflammation has also been implicated in oral carcinogenesis through mechanisms involving genomic instability, immune dysregulation, and EMT [15,16].

ZEB1 is a key EMT-regulating transcription factor that promotes cellular motility, invasion, and disease progression by suppressing epithelial markers and enhancing mesenchymal characteristics [17]. Its overexpression has been associated with poor prognosis, metastasis, and therapeutic resistance in several malignancies, including OSCC [18]. However, comparative data regarding salivary ZEB1 levels among healthy individuals, periodontitis patients, and OSCC patients remain limited [19].

In the present study, the mean age was greater in the periodontitis group compared with the healthy and OSCC groups and was associated with greater periodontal destruction. Similar observations have been reported by Park et al., who demonstrated an association between advancing age and increased prevalence and severity of periodontitis [20]. Likewise, Warnakulasuriya identified age as an important risk factor for OSCC development [21]. These findings support the role of aging as a contributing factor to both chronic periodontal inflammation and oral carcinogenesis.

A higher proportion of males was observed in both the periodontitis and OSCC groups. Previous studies by Albandar et al. and Grossi et al. similarly reported a higher prevalence and severity of periodontal disease among males [22,23]. In addition, Pérez-Sayáns et al. observed a higher incidence of OSCC among males, particularly in populations with increased exposure to tobacco and alcohol-related risk factors [24]. Nevertheless, the gender distribution in the present study was not statistically significant, which is consistent with recent evidence suggesting a gradual narrowing of the gender gap in oral cancer incidence [25].

The clinical periodontal parameters evaluated in this study, including OHI-S, PPD, CAL, and Russell’s Periodontal Index, were significantly higher in the periodontitis group, indicating greater periodontal tissue destruction. These findings are consistent with previous reports demonstrating a strong association between poor oral hygiene, increased periodontal parameters, and disease severity [26-28].

A notable finding of the present study was the significantly elevated salivary ZEB1 levels in periodontitis patients compared with healthy individuals. This observation suggests a possible association between ZEB1 expression and periodontal inflammation. Similar findings have been reported by Qian et al., who demonstrated increased ZEB1 expression in periodontal tissues [29]. Furthermore, previous studies have shown that enhanced ZEB1 expression may be associated with inflammatory responses, EMT activation, and altered periodontal ligament stem cell function, supporting its potential role in periodontal tissue breakdown [30,31].

EMT-related processes have been proposed as an important mechanism in the pathogenesis of periodontitis, with chronic inflammation stimulating the expression of transcription factors such as ZEB1. Consequently, increased local expression of ZEB1 within inflamed periodontal tissues, together with its release from damaged epithelial and connective tissue cells into the oral environment, may contribute to elevated salivary ZEB1 levels observed in individuals with periodontitis [29,30].

The highest salivary ZEB1 levels were observed in the OSCC group. Elevated ZEB1 expression has been associated with EMT-mediated tumor progression, reduced epithelial adhesion, enhanced invasion, and metastatic potential. The present findings are in agreement with Panchannavar et al., who reported increased ZEB1 expression in oral potentially malignant disorders and OSCC [32]. Although the study groups differed significantly in age, ANCOVA demonstrated that study group remained significantly associated with salivary ZEB1 levels after adjustment for age and gender, whereas neither age nor gender independently influenced salivary ZEB1 concentrations.

The concurrent elevation of salivary ZEB1 levels observed in both periodontitis and OSCC groups suggests that ZEB1 may be associated with inflammatory and EMT-related processes present in these conditions. These findings are consistent with the meta-analysis by Ma et al., which reported an association between periodontitis and an increased risk of oral cancer [33]. However, due to the cross-sectional design, no causal relationship or shared biological pathway between periodontitis and OSCC can be inferred from the present findings. Rather, it suggests that ZEB1 may represent a common biomarker associated with both disease processes. Further mechanistic and longitudinal studies are required to determine its biological and clinical significance.

Despite these findings, several limitations should be acknowledged, including the single-center cross-sectional design, relatively small sample size, age- and gender-unmatched cohorts, absence of longitudinal follow-up, lack of tissue-level evaluation of ZEB1 expression, and inability to assess variations across TNM stages, histopathological grades, and periodontal disease severities. The cross-sectional nature of the study permits identification of associations only and precludes conclusions regarding causality. Formal examiner calibration and intra-examiner reliability assessment were not performed, which may have introduced measurement variability. In addition, unstimulated salivary flow rate was not measured, ZEB1 levels were not normalized to total protein concentration, biomarker assessment was based on a single saliva sample, and minor blood contamination could not be completely excluded. Tobacco use, alcohol consumption, diet, socioeconomic status, diabetes status, and unidentified systemic conditions were not separately evaluated and may have acted as confounding factors. Furthermore, as only a single biomarker was evaluated, a definitive biological relationship between periodontitis and OSCC cannot be established. Future studies employing larger multicenter cohorts, random sampling strategies, repeated saliva sampling over time, and comprehensive control of potential confounding factors are warranted to validate the present findings. In addition, incorporation of multiple biomarkers, detailed analytical validation, and longitudinal study designs may help improve biomarker reliability, account for intraindividual variability, and further elucidate the role of ZEB1 in periodontal disease progression and oral carcinogenesis.

## Conclusions

Salivary ZEB1 levels were significantly elevated in both periodontitis and OSCC groups, with the highest expression observed in OSCC patients. These findings suggest the involvement of ZEB1 in chronic periodontal inflammation, tissue destruction, tumor progression, and EMT. The observed association between periodontitis and OSCC may be mediated through shared inflammatory and oncogenic pathways involving ZEB1. Overall, salivary ZEB1 shows considerable promise as a non-invasive biomarker for the diagnosis and monitoring of periodontal disease and OSCC.
